# Pollen flow and effects of population structure on selfing rates and female and male reproductive success in fragmented *Magnolia stellata* populations

**DOI:** 10.1186/1472-6785-13-10

**Published:** 2013-03-22

**Authors:** Suzuki Setsuko, Teruyoshi Nagamitsu, Nobuhiro Tomaru

**Affiliations:** 1 Department of Forest Genetics, Forestry and Forest Products Research Institute, Tsukuba, Ibaraki 305-8687, Japan; 2 Laboratory of Forest Ecology and Physiology, Graduate School of Bioagricultural Sciences, Nagoya University, Nagoya 464-8601, Japan

**Keywords:** Conservation, Fragmentation, Gene flow, Geitonogamy, Insect pollination, Landscape, Magnoliaceae, Paternity analysis, Pollen dispersal, Seed production

## Abstract

**Background:**

Fragmentation of plant populations may affect mating patterns and female and male reproductive success. To improve understanding of fragmentation effects on plant reproduction, we investigated the pollen flow patterns in six adjacent local populations of *Magnolia stellata*, an insect-pollinated, threatened tree species in Japan, and assessed effects of maternal plant (genet) size, local genet density, population size and neighboring population size on female reproductive success (seed production rates), and effects of mating distance, paternal genet size, population size and separation of populations on male reproductive success.

**Results:**

The seed production rate, i.e. the proportion of ovules that successfully turned into seeds, varied between 1.0 and 6.5%, and increased with increasing population size and neighboring population size, and with decreasing maternal genet size and local genet density. The selfing rate varied between 3.6 and 28.9%, and increased with increasing maternal genet size and with declining local genet density. Male reproductive success increased with increasing paternal genet size, and decreased with increasing mating distance and separation of population. Pollen flow between the populations was low (6.1%) and highly leptocurtic.

**Conclusions:**

Our results indicate that habitat fragmentation, separation and reduced size of populations, affected mating patterns and reproductive success of *M*. *stellata*. Local competition for pollinators and plant display size were likely to alter the reproductive success.

## Background

Fragmentation disrupts continuous populations, resulting in smaller, separated populations. The fragmentation of plant populations may decrease seed production and outcrossing rates due to limitations of mates and pollinators in the remaining, fragmented populations, potentially reducing reproductive success [1,2]. Thus, the reproductive success of parent plants and fitness of their offspring may be reduced [3-6]. Fragmentation may also lead to reductions in the frequency of gene flow among populations and genetic variation within populations due to genetic drift [7]. Among plant taxa, woody plant species are relatively resistant to reductions in reproductive success and genetic variation due to habitat fragmentation owing to the longevity of individuals, relatively high local genetic diversity and extensive gene flow [8,9]. However, recent meta-analyses suggest that fragmentation is likely to reduce the genetic variation of woody plant species as much as that of herbaceous species [10], and to affect reproductive success of trees as much as that of other life forms [7].

It is especially difficult to predict the effects of population fragmentation on mating patterns and seed production of animal-pollinated trees, since the abundance and behavior of pollinators may change as the size and isolation of their populations change [11,12]. Reductions in the size of tree populations are expected to decrease the abundance of resident pollinators and the attraction of pollinators migrating between populations [13]. Higher local densities of flowering trees may attract disproportionately more pollinators, resulting in higher pollination frequencies [14]. On the other hand, increases in local tree density may increase local competition for pollinators, as observed in large populations of insect-pollinated shrubs [15]. At the individual level, the size of trees, which is related to floral display size and resource availability, is also likely to affect pollination rates and seed production.

Particularly in mixed-mating trees, the selfing rate is likely to increase as the availability of mates and pollinators decreases in small, fragmented populations [2,7,16]. Geitonogamous selfing (self-pollination between flowers within individuals), which is prevalent in trees, also tends to increase in large trees that produce abundant flowers and at sites with low local floral density and limited mate availability [17,18]. Selfed offspring of most outcrossing trees with high degrees of inbreeding depression [19,20] have low survival and growth rates, and consequently low fitness. Population fragmentation is also likely to alter patterns of pollen-mediated gene flow (pollen flow). Forest fragmentation can either impede or facilitate pollen flow between tree populations [21-23], because landscape elements between the remaining forests may have either positive or negative effects on pollinator transfer [24]. Rather than hindering pollen flow, fragmentation and reductions in tree density frequently result in increased pollination distances [25]. The rates of immigrant pollen flow from outside often increases in fragmented populations, except in extremely isolated populations [22,25-27]. Furthermore, not only immigration but also emigration of pollen, i.e. pollen donation from one population to another, may play important roles in the maintenance of genetic connectivity among populations [28]. Hence, measuring both the direction and frequency of pollen flow between populations is necessary to evaluate effects of population fragmentation on mating patterns.

An explicit method to clarify the direction of pollen flow between populations, and quantify the composition of contemporary pollen flow, is paternity analysis of seeds collected from known mothers to determine the origin of pollen that fertilized seeds. It was first applied by Adams and Birkes [29,30] in attempts to simultaneously estimate pollen flow and factors related to male fertility, such as distances to mother trees and the degree of floral synchrony. This approach was extended by Burczyk et al. [31], who constructed a neighborhood model to utilize genetic marker data from diploid offspring, and Klein et al. [32], who proposed a new spatially explicit model to estimate jointly the variance of male fecundity and pollen dispersal kernels. However, in fragmented tree populations, mating patterns may depend not only on individual properties, such as the tree size and inter-tree distance, but also population properties, such as the size and separation of populations. Hence, models including factors at both individual and population levels are required to estimate determinants of pollen flow patterns and male reproductive success.

In this study, we assessed pollen flow and assessed selfing rates and female and male reproductive success in six fragmented populations of a threatened tree species, *Magnolia stellata*. This species produces insect-pollinated flowers, it has a mixed mating system with substantial amounts of selfed seeds, and its seed production is often pollen-limited [33,34]. Thus, the species is suitable for investigating effects of population fragmentation on selfing rates and reproductive success. First we assessed seed production rates in the studied populations to obtain estimates of female reproductive success, and then conducted paternity analysis of seeds collected from the trees. Next, we examined the selfing rates, from the assigned paternity data. Then, we estimated male reproductive success and pollen dispersal from the results of the paternity analysis. Finally, we evaluated the effects of genet size, local genet density, population size and neighboring population size as a indicator of population isolation on female reproductive success and selfing rates, and the effects of mating distance, genet and population size and geographical separation of the populations on male reproductive success in the populations.

## Methods

### Study species

*Magnolia stellata* Maxim. [35] (synonym, *M*. *tomentosa* Thunb.; [36]) is a deciduous tree of the Magnoliaceae. The species is endemic to the area around the Ise Bay of Central Japan and is now considered near threatened (NT) in the Japanese Red List [37]. Habitats of this species are swampy places, such as streamsides and marshes, in the uppermost parts of valleys [38]. The species reaches heights up to 10 m. It blossoms in early spring, forming protogynous, insect-pollinated flowers. The mean flowering duration for an individual tree is 13.2 days, and the mean total flowering duration of the individual flower and the durations of the female, transitionary and male phases of individual flowers are reportedly 10.3, 4.1, 0.7 and 5.5 day, respectively [39]. The main flower visitors are small beetles [40], as in many other *Magnolia* species [41-44]. Each flower can produce one fruit. The fruits have ca. 30 to 40 carpels, and each carpel usually has two ovules and can produce at most two red seeds. Seeds are dispersed by both gravity [45] and birds, such as the brown-eared bulbul (*Hypsipetes amaurotis*) and Japanese thrush (*Turdus cardis*) (T. Kimura et al. unpublished data).

### Study area

The research site is located in the Kaisho Forest, near Nagoya City, Aichi Prefecture, Japan (35˚ 11’ 25” N, 137˚ 06’ 55” E). This area supports a secondary forest that is mainly composed of *Pinus densiflora*, *Ilex pedunculosa*, *Quercus serrata* and *Clethra barvinervis*[46]. The potential natural vegetation of this area is evergreen broad-leaved forest, but the forest is now mainly dominated by deciduous trees because people utilized the trees for fuel wood for a long time in the past. However, since people stopped using the wood for fuel, evergreen broad-leaved trees have been increasing via secondary succession.

**Figure 1:**
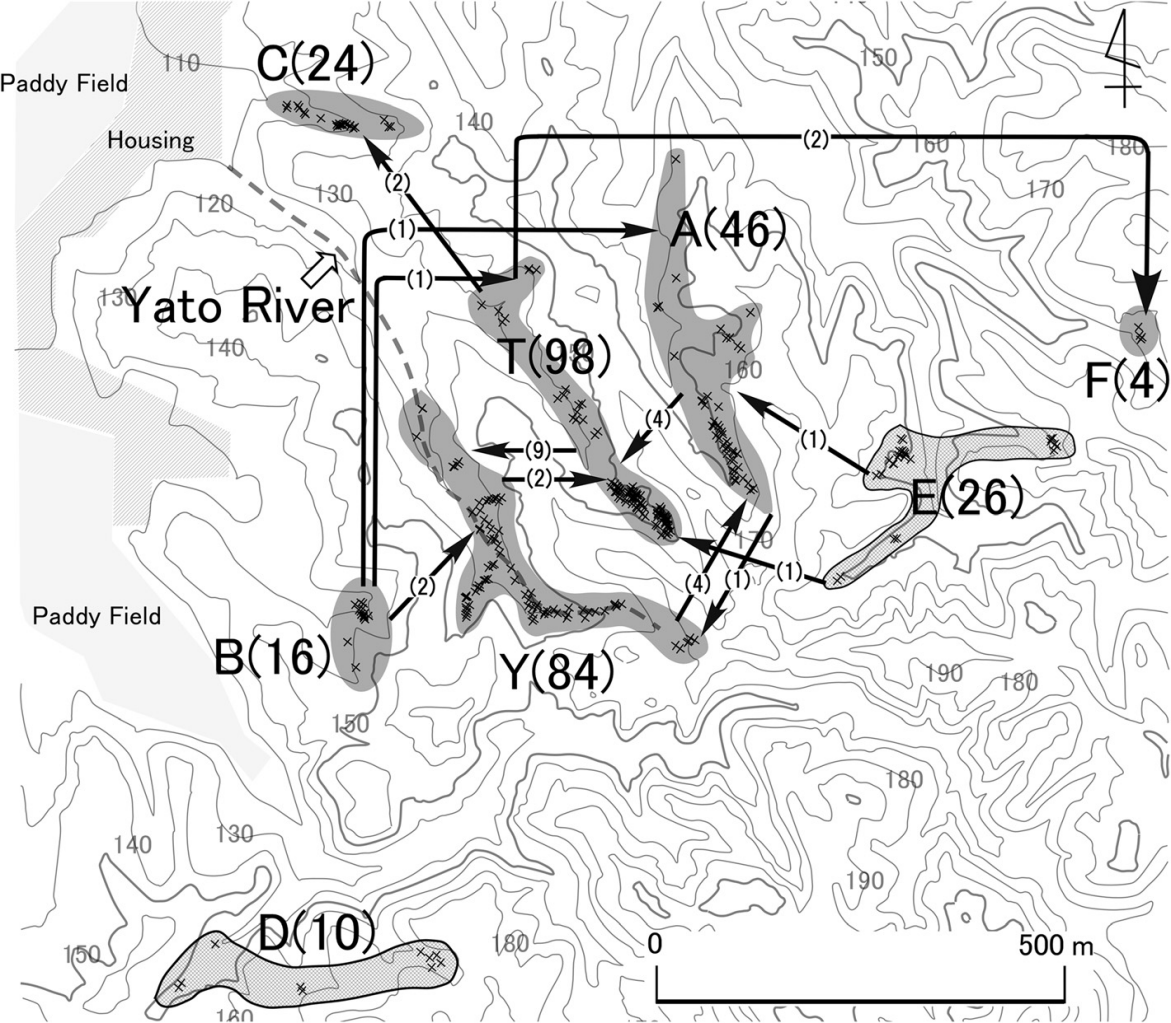
**Spatial distribution of *Magnolia stellata *populations and genets examined in this study.** The letters and numbers in parenthesis indicate the designations of populations and numbers of adult genets (i.e. population size), respectively. Crosses indicate the locations of genets. Gray (Y, T, A, B, C, F) and meshed areas (D, E) indicate locations of populations in which seeds were and were not sampled, respectively. Arrows represent pollen flow and the numbers by the arrows indicate the numbers of pollen migration events.

We define ‘a population’ here as a group of genets in the uppermost part of one of the valleys in the area, each of which is separated from adjacent populations by ridges (Figure 1). Eight *M*. *stellata* populations (designated Y, T, A, B, C, D, E and F) in the southwestern part of the Kaisho Forest were surveyed. The locations and designations of these populations are shown in Figure 1. No other population is present in or around the study area and the nearest population outside the study area is about 1 km north of Population C.

### Determination of adult genets

Since *M*. *stellata* genets usually consist of several ramets, due to sprouting and layering [45], different genets were distinguished in previous studies based on the connections between ramets above the ground and multilocus nuclear microsatellite (nSSR) genotypes [38,45]. In one of the previous studies we also surveyed all genets that flowered at least once from 2002 to 2004 (defined as adult genets) in the eight populations, and recorded the spatial coordinates and diameter at breast height (DBH) of the largest stem of each adult genet in 2007 [39].

### Measures of female reproductive success

In March 2005, we selected three to 14 genets (8.3 on average and 50 in total) from six populations (Y, T, A, B, C and F) and marked 11 to 96 individual flowers (33 on average and 1648 in total) of each genet (Table 1). In August 2005, we sampled mature fruits that developed from the marked flowers. The number of ovules in the marked flowers of each selected genet was estimated from the number of carpels in sampled fruits of the genet, assuming that flowers and fruits had equal numbers of carpels, and that each carpel had two ovules [numbers of ovules in flowers = numbers of carpels in fruits × 2]. The fruit set percentages [(the number of sampled fruits from each genet / the number of marked flowers of each genet) × 100], and seed set [(the total number of filled seeds in sampled fruits of each genet/ the total number of ovules in marked flowers of each genet) × 100] were calculated for every selected genet. As a measure of female reproductive success, the percentage of ovules that developed into filled seeds (seed production rate = fruit set × seed set × 100) was calculated for every selected genet.

**Table 1:** Seed production rate, results of paternity analysis, immigration and selfing rates per tree per population

**Population (population size)**	**Seed parents**	**DBH (cm)**	**No. flower marked**	**Seed production rate (%)**	**No. samples for paternity analysis**	**Immigration rate by pollen (%)**		**Pollen source population**	**Selfing rate (%)**	
Y (84)	Y4	5.4	-	-	4	0.0	(0)	-	0.0	(0)
	Y5	6.0	-	-	17	11.8	(2)	T (2)	0.0	(0)
	Y20	6.3	-	-	19	0.0	(0)	-	0.0	(0)
	Y56	6.0	46	5.2	6	0.0	(0)	-	100.0	(6)
	Y83	6.2	73	0.6	13	7.7	(1)	T (1)	0.0	(0)
	Y90	9.0	-	-	6	0.0	(0)	-	83.3	(5)
	Y91	9.2	-	-	17	5.9	(1)	B (1)	0.0	(0)
	Y98	5.3	-	-	11	0.0	(0)	-	0.0	(0)
	Y129	4.4	20	1.6	3	0.0	(0)	-	33.3	(1)
	Y136	1.4	-	-	3	66.7	(2)	A(1), B(1)	0.0	(0)
	Y137	3.2	90	3.2	11	27.3	(3)	T (3)	27.3	(3)
	Y141	2.8	12	7.6	15	0.0	(0)	-	0.0	(0)
	Y145	5.7	96	6.0	12	16.7	(2)	T (2)	41.7	(5)
	Y150	3.7	17	4.5	17	5.9	(1)	T (1)	5.9	(1)
	Y158	5.7	54	7.2	1	0.0	(0)	-	100.0	(1)
	Y160	6.4	-	-	9	0.0	(0)	-	66.7	(6)
	Y161	3.8	29	4.0	3	0.0	(0)	-	33.3	(1)
	Y175	4.6	18	3.5	-	-	-	-		
Mean (Total)		5.28	45.50 (455)	4.33	9.82 (167)	8.34	(12)	T (9), A(1), B(2)	28.91	(29)
T (98)	T2	5.7	-	-	14	14.3	(2)	Y(1), A(1)	0.0	(0)
	T17	7.7	71	0.7	6	0.0	(0)	-	0.0	(0)
	T24	3.8	25	4.7	2	0.0	(0)	-	0.0	(0)
	T25	3.9	39	1.0	7	0.0	(0)	-	0.0	(0)
	T26	5.3	22	4.4	8	0.0	(0)	-	0.0	(0)
	T27	3.7	35	10.1	15	0.0	(0)	-	0.0	(0)
	T29	4.1	42	6.4	6	16.7	(1)	A (1)	0.0	(0)
	T52	2.5	42	4.6	25	0.0	(0)	-	0.0	(0)
	T53	2.8	35	2.9	16	6.3	(1)	A (1)	6.3	(1)
	T54	2.5	38	2.2	8	12.5	(1)	A (1)	25.0	(2)
	T77	3.7	14	0.0	-	-	-	-	-	-
	T91	5.1	32	26.0	1	0.0	(0)	-	0.0	(0)
	T92	6.5	33	3.4	10	20.0	(2)	Y(1), B(1)	50.0	(5)
	T99	2.4	22	3.0	10	10.0	(1)	E (1)	0.0	(0)
	T100	3.7	63	5.4	9	0.0	(0)	-	0.0	(0)
Mean (Total)		4.22	36.64 (513)	5.35	9.79 (137)	5.69	(8)	Y(2), A(4), B(1), E(1)	5.80	(8)
A (46)	A1	5.9	32	1.2	1	0.0	(0)	-	0.0	(0)
	A2	7.5	18	5.5	4	0.0	(0)	-	25.0	(1)
	A3	10.4	22	1.4	2	50.0	(1)	Y (1)	0.0	(0)
	A18	4.0	18	7.4	9	11.1	(1)	Y (1)	0.0	(0)
	A22	7.7	21	4.6	11	9.1	(1)	B (1)	0.0	(0)
	A24	6.7	17	10.7	18	5.6	(1)	Y (1)	0.0	(0)
	A25	8.4	20	14.7	22	9.1	(2)	Y(1), E(1)	0.0	(0)
Mean (Total)		7.22	21.14 (148)	6.50	9.57 (67)	12.12	(6)	Y(4), B(1), E(1)	3.57	(1)
	B2	5.0	46	4.7	9	0.0	(0)	-	0.0	(0)
	B9	5.0	71	0.3	-	-	-	-	-	-
	B14	5.0	24	1.0	11	0.0	(0)	-	18.2	(2)
	B18	5.9	42	0.8	6	0.0	(0)	-	33.3	(2)
	B31	3.8	18	0.2	4	0.0	(0)	-	50.0	(2)
	B32	4.8	16	0.0	-	0.0	(0)	-	-	-
	B33	5.5	21	0.0	-	-	-	-	-	-
	B35	3.1	11	0.0	-	-	-	-	-	-
	B41	5.8	22	1.7	-	-	-	-	-	-
Mean (Total)		4.87	30.11 (271)	0.98	7.50 (30)	0.00	(0)	-	25.38	(6)
C (24)	C1	13.0	30	0.3	13	7.7	(1)	T (1)	38.5	(5)
	C21	7.1	25	5.4	10	10.0	(1)	T (1)	20.0	(2)
	C25	7.3	19	5.0	2	0.0	(0)	-	0.0	(0)
	C28	4.4	17	6.3	21	0.0	(0)	-	0.0	(0)
	C34	8.2	13	13.8	19	0.0	(0)	-	15.8	(3)
	C36	6.8	14	2.4	8	0.0	(0)	-	12.5	(1)
	C43	2.6	12	3.3	10	0.0	(0)	-	10.0	(1)
Mean (Total)		7.04	18.57 (130)	5.21	11.86 (83)	2.53	(2)	T (2)	13.82	(12)
F (4)	F1	8.4	23	0.4	2	0.0	(0)	-	0.0	(0)
	F2	8.4	23	0.1	2	0.0	(0)	-	50.0	(1)
	F4	5.4	22	2.5	5	40.0	(2)	T (2)	0.0	(0)
Mean (Total)		7.42	22.67 (68)	1.03	3.00 (9)	13.33	(2)	T (2)	16.67	(1)

### Sampling and germination test for paternity assignment

To increase the number of samples for paternity analysis, we also sampled fruits from nine additional genets that we did not survey for female reproductive success (eight in Population Y and one in T). The fleshy pulp of seeds was removed from the sampled fruits, and the seeds were stored in water at 5°C for 48 weeks. Then, 30 seeds from each genet (or all seeds from genets yielding less than 30 seeds) were sown on damp filter paper. The seeds were then placed in an incubator under diurnal temperature cycles of 12 h at 5°C and 12 h at 25°C for about six months to induce germination. The numbers of seedlings that had germinated at the end of this time ranged from 0 to 25 per genet, nine to 167 per population, and 493 seedlings in total were obtained (Table 1).

### DNA extraction and microsatellite genotyping

Genomic DNA was extracted from leaves of adult genets and seedlings (hereafter referred to as offspring) using a modified CTAB (Cetyltrimethylammonium bromide) method [47] with further minor modifications. Genotypes of the 493 offspring were determined using 10 SSR markers, namely *M6D8* developed for *M*. *obovata*[48], and nine previously developed for *M*. *stellata*: *stm0148*, *stm0184*, *stm0191*, *stm0222*, *stm0223*, *stm0251*, *stm0334*, *stm0353* and *stm0423*[49]. Genotypes at the same loci of all the 306 adult genets in the eight populations had already been determined in a previous study [38].

### Paternity assignment

High levels of genetic diversity (*H*_E_ = 0.773 ± 0.019) have been observed in the adult genets of all the populations [38]. Based on fixation indices (*F*_IS_), no significant deviation from Hardy-Weinberg equilibrium was found at any loci in any populations. No significant linkage disequilibrium between loci was observed for any population, except at one pair of loci in one population. Therefore, the loci seemed to be independent and had few null alleles, and the exclusion probability for the second parent was 0.99992. Detailed summaries of the data pertaining to these findings are available in Setsuko et al. [38].

Parentage analyses were performed based on the multilocus SSR genotypes of the offspring and candidate parents (i.e. adult genets) using CERVUS version 3.0 [50] with maximum-likelihood algorithms [51]. The simulation parameters were as follows: 10,000 cycles, 306 candidate parents, 0.95 as the proportion of candidate parents sampled, 0.997 (calculated from the data) as the proportion of loci typed, 0.01 as the rate of typing errors, 95% for the strict confidence level and 80% for the relaxed confidence level. For the proportion of candidate parents sampled, we were confident that we sampled all extant candidate parents within the populations. However, we did not conduct a flowering census in 2005 (the year seeds were sampled), so it is possible that a few adult genets that had not identified as flowering genets in the censuses from 2002 to 2004 may have flowered in 2005. Therefore, we set this parameter at 0.95. We allowed selfing in the parentage testing, because *M*. *stellata* is self-compatible, setting the selfing rate at 0.20 based on our previous data [52]. According to the assigned paternity data, we categorized the offspring as derived from selfing, outcrossing within the study area, and outcrossing with a paternal parent that was not present in the study area. We defined the selfing rate as the number of selfed offspring divided by the number of examined offspring from each genet.

### Factors influencing the female reproductive success and selfing rate

To investigate the factors that influence the female reproductive success and selfing rate, we applied a GLM with a binomial error distribution and a logistic-link function using R 2.11.1 [53]. We defined genet size as the basal area (cm^2^) of the largest stem of each adult genet at breast height (π× (DBH of the largest stem of genet/2)^2^). As explanatory variables in the GLM, we chose maternal genet size (genet size of a focal maternal genet), local genet density (summed size of adult genets within a certain radius, *x* in meters, of the focal maternal genet), population size (summed size of adult genets in the focal maternal genet’s population) and neighbouring population size (sum of neighbouring population size within a 300 m radius from the focal maternal genet) as a indicator of population isolation. We have previously shown that the size of maternal genets affects their floral display size and thus attractiveness to pollinators [39,40]. Therefore, we used summed genet sizes to indicate the local genet density and population size, rather than numbers of adult genets, in order to evaluate effects of both the number and size of individual genets simultaneously. To determine the most appropriate radius for neighbouring genets, radii with 5 m increments from 5 to 50 m were tested in the GLMs. We set the upper radius at 50 m since most pollen flow occurs within 50 m in this species [38]. Neighboring populations were defined as the populations that including at least one individual within the radius 300 m from the focal maternal genet except for the focal maternal population. The radius for neighboring population was set as 300 m since it can cover the adjacent populations from each focal population. To select models for estimating parameters for the explanatory variables we calculated Akaike’s Information Criterion (AIC) values [54]. Models with lower AIC values have higher likelihoods and smaller numbers of parameters than alternative models.

### Estimation of pollen dispersal parameters and factors affecting male reproductive success

Pollen dispersal events identified by paternity analyses were used to estimate a pollen dispersal kernel characterizing pollen movements within our study area, in terms of an exponential power function [55]. Using this function, the probability of pollen dispersal *d* over distance *r* (m) was defined as:

$$d=\frac{a^{2}b}{2\pi \Gamma \left(2/b\right)}\exp\left(-{\left[\mathrm{ar}\right]}^{b}\right),$$

where Γ is the gamma function, and *a* and *b* are scale and shape parameters, respectively. The mean dispersal distance is expressed as Γ (3/*b*)/*a*Γ (2/*b*) [56]. This function has been more frequently used than Weibull, geometric, Student’s *t* and other functions because various dispersal patterns can be expressed using solely the shape parameter of the exponential power function, such as an exponential distribution (*b* = 1), normal distribution (*b* = 2), fat-tailed (*b* < 1) or thin-tailed (*b* > 1), leptokurtic (*b* < 2) or platykurtic (*b* > 2) distributions [56].

We examined the male reproductive success based on the dispersal kernel. For maternal genet *i* = {1, 2, …, *M*}, the number of seeds *n*_*ij*_ (*i* ≠ *j*) sired by male genet *j* = {1, 2, …, *N*} is assumed to follow a multinomial distribution with the probability *p*_*ij*_ and sample size $\sum_{k=1\left(k\neq i\right)}^{N-1}n_{\mathrm{ik}}$. The probability *p*_*ij*_ (*i* ≠ *j*) that male genet *j* sired seeds of maternal genet *i* was determined using parameters of pollen dispersal *a*, *b* and male reproductive success *f*_*ij*_ (*i* ≠ *j*) from male genet *j* to maternal genet *i* in the form:

$$p_{\mathrm{ij}=}\frac{f_{\mathrm{ij}}\;\text{exp}\;\left(-{\left[ar_{\mathrm{ij}}\right]}^{b}\right)}{\sum_{k=1\left(k\neq i\right)}^{N-1}f_{\mathrm{ik}}\;\exp\;\left(-{\left[ar_{\mathrm{ik}}\right]}^{b}\right)},$$

where *r*_*ij*_ (*i* ≠ *j*) is the distance (m) between maternal genet *i* and male genet *j*. Male reproductive success *f*_*ij*_ was determined from the relative size *s*_*ij*_ of male genet *j* to maternal genet *i* (*s*_*ij*_ = genet size of *j* / genet size of *i*, relative genet size), the relative size *t*_*ij*_ of the population of male genet *j* to the population of maternal genet *i* (*t*_*ij*_ = population size of genet *j* / population size of genet *i*, relative population size) and the geographic separation *u*_*ij*_ of populations of the maternal genet *i* and male genet *j* (*u*_*ij*_ = 0 when the maternal and male genets belonged to the same population, and *u*_*ij*_ = 1 when they belonged to different populations, separation of population) in the form:

$$f_{\mathrm{ij}}=s_{\mathrm{ij}}{}^{a}t_{\mathrm{ij}}{}^{\beta }\;\exp\left(\gamma \mu_{\mathrm{ij}}\right),$$

where α, β and γ are parameters of *s*_*ij*_, *t*_*ij*_ and *u*_*ij*_, respectively. The mating likelihood function for *M* maternal genets and *N* male genets was expressed as:

$$L(n,r,s,t,u\;|\;a,b,\alpha ,\beta ,\gamma )=\prod_{i=1}^{M}\prod_{j=1\left(j\neq i\right)}^{N-1}p_{\mathrm{ij}}{}^{n_{\mathrm{ij}}}.$$

The posterior probability distributions were derived from the likelihood and non-informative prior probability distributions (gamma distributions with mean 1 and variance 1,000 for *a* and *b*, and normal distributions with mean 0 and variance 1,000 for α, β and γ).

Posterior distributions of the parameters were computed as conditional distributions that were updated based on the *n*_*ij*_, *r*_*ij*_, *s*_*ij*_, *t*_*ij*_, and *u*_*ij*_ data. Initial values (*a* = *b* = 1, α = β = γ = 0) were first defined and then updated, to fit the data relating to the probability *p*_*ij*_ that male genet *j* sired seeds of maternal genet *i*, in three chains of MCMC sampling implemented in JAGS software using the rjags package in R2.11.1 [57]. Each chain was run for 20,000 iterations with parameter values recorded every 20 iterations after a burn-in period of 2,000 iterations. MCMC convergence after all iterations in the three chains for the parameters was visualized by the coda package, and confirmed by R-hat < 1.1 using the function gelman.diag. The parameter estimates, the median and range between 2.5 and 97.5 percentiles, were obtained from the 2,700 MCMC samples.

## Results

### Female reproductive success

The mean seed production rate for each population ranged from 0.98% (population B) to 6.50% (population A) and was 4.00% for the six populations on average (Table 1). According to the AIC and ΔAIC values, the model providing the best explanation for the variation in the seed production rate included four explanatory variables: maternal genet size, local genet density within a 50 m radius, population size and neighboring population size (Table 2). The estimated parameters of these variables were positive for population size and neighboring population size and negative for maternal genet size and local genet density (Table 3), indicating that female reproductive success increased as the population size and neighboring population size increased and as the maternal genet size and the local genet density decreased.

**Table 2:** **The intercept and parameters included in the best and other models (according to Akaike’s Information Criterion, AIC) explaining the female reproductive success and selfing rate of *Magnolia stellata *offspring**

**Model rank**	**Ovule survival rate**			**Selfing rate**		
	**Model**	**AIC**	**ΔAIC**	**Model**	**AIC**	**ΔAIC**
1	*c, maternal genet size, local density (50 m), population size, neighboring population size*	15766.1	0.0	*c, maternal genet size, local density (25 m)*	299.5	0.0
2	*c, maternal genet size, local density (50 m), neighboring population size*	15776.5	10.4	*c, maternal genet size, local density (25 m), population size*	300.8	1.3
3	*c, maternal genet size, local density (40 m), population size, neighboring population size*	15791.8	25.7	*c, maternal genet size, local density (25 m), neighboring population size*	301.3	1.8
4	*c, maternal genet size, local density (45 m), population size, neighboring population size*	15799.9	33.8	*c, local density (25 m)*	301.7	2.2
5	*c, local density (40 m), pulation size, neighboring population size*	15805.4	39.3	*c, maternal genet size, local density (30 m)*	301.8	2.3

Abbreviations: ΔAIC, the difference in AIC between the model considered and the most parsimonious model; *c*, intercept. Numbers in the parentheses after *local density* are the radius used to calculate the local genet density.

**Table 3:** **Fixed explanatory variables of the generalized linear models that best explained female reproductive success, and the selfing rate of *Magnolia stellata *populations, selected according to Akaike’s information Criterion (AIC)**

**Response variables**	**Explanatory variables**	**Coefficients**	***S.E.***	***p *value**
Ovule survival rate	*c*	−4.20200	0.05343	<0.001
	*maternal genet size*	−0.00256	0.00074	<0.001
	*local density (50 m)*	−0.00056	0.00004	<0.001
	*population size*	0.00055	0.00003	<0.001
	*neighboring population size*	0.00013	0.00001	<0.001
Selfing rate	*c*	−1.21029	0.47454	<0.05
	*maternal genet size*	0.12485	0.06044	<0.05
	*local density (25 m)*	−0.00534	0.00105	<0.001

Abbreviations: *c*, intercept. Numbers in the parentheses after *local density* are the radius used to calculate the local genet density.

### Paternity assignment

Pollen parents of all 493 offspring examined here were identified at the 95% confidence level, thus no offspring with a pollen parent outside the study area was found. Of the 493 offspring, 57 (11.56%) were derived from selfing (Table 1), and pollen parents of 436 outcrossed progeny were assigned to 115 out of 306 candidates (37.58%) within the study area.

### Selfing rate

No selfing was detected for 30 genets, but the selfing rates of the other 22 ranged from 5.88 to 100.00% (Table 1). The model providing the lowest AIC value for the variations in the selfing rate included two explanatory variables (maternal genet size and local genet density within a 25 m radius) (Table 2). The estimated parameter for the local genet density was negative (Table 3), indicating that the selfing rates were higher for genets with lower densities of neighboring genets. The estimated parameter for the maternal genet size was positive, suggesting that larger genets have higher selfing rates.

### Pollen flow and male reproductive success

Of the 436 outcrossed progeny, 30 originated from mating between genets in different populations (6.88%), and 406 from mating between genets in the same populations (93.12%) (Table 1, Additional file 1). Among pollen flow events detected between populations, the most frequent were from T to Y, A to T and Y to A (nine, four and four mating events, respectively; Figure 1, Additional file 1). All six populations used in this analysis were connected by pollen flow, but the central three populations (A, T and Y) were both donors and recipients of the observed pollen flow, while the peripheral populations were either donors (population B) or recipients (populations C and F) (Figure 1).

**Figure 2:**
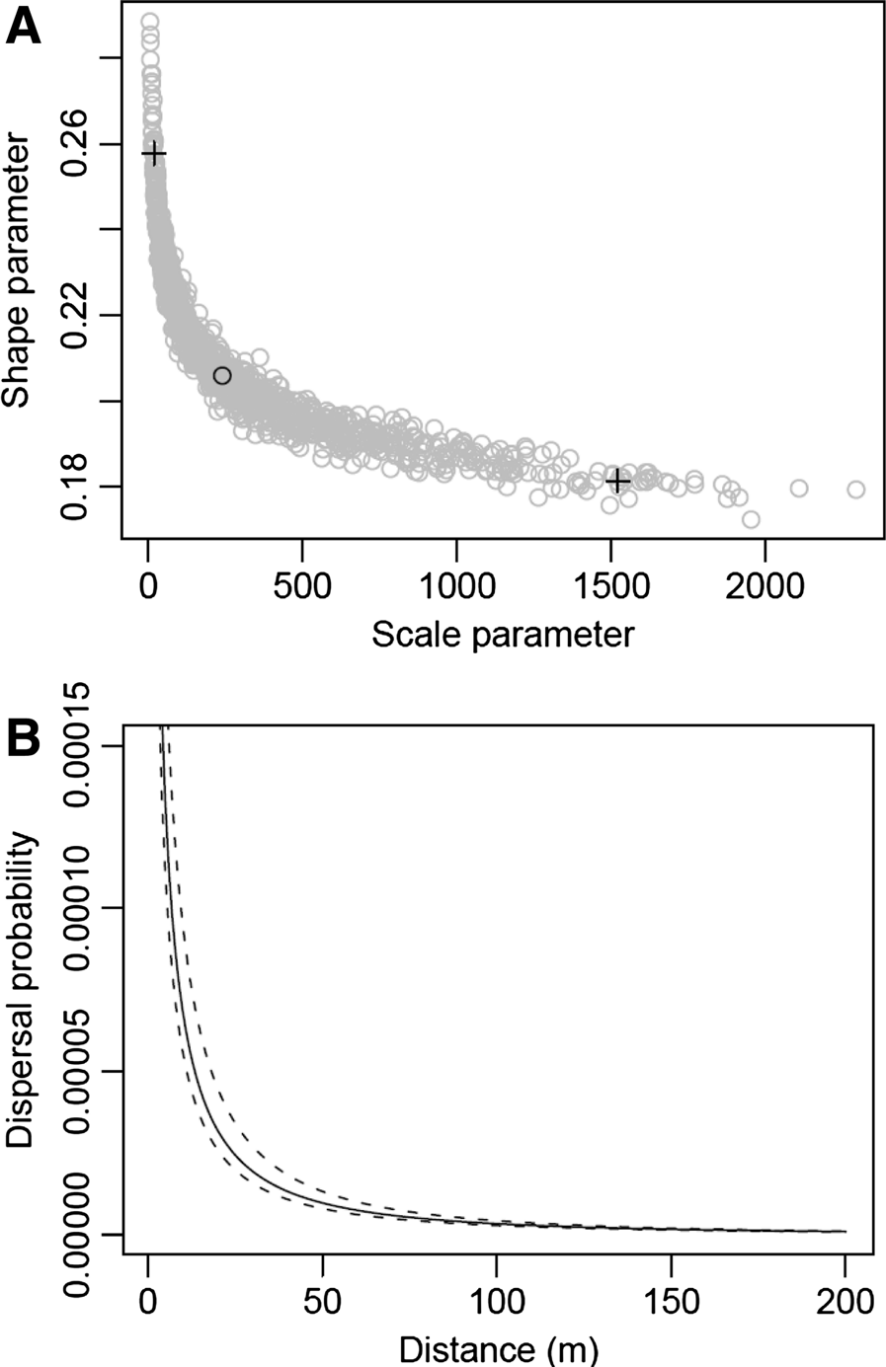
**A) Distributions of scale (*a*) and shape parameters (*b*) obtained by MCMC sampling.** The black circle indicates the median of the two parameters and plus signs indicate upper and lower limits of their 95% credible intervals. **B**) Estimated pollen dispersal curve (solid line) derived from modeling pollen dispersal with male reproductive success, when medians of the scale and shape parameters are applied, and upper and lower limits of the 95% credible interval obtained by MCMC sampling (dashed lines).

On the basis of the observed pollen flow pattern, posterior distributions of the two pollen dispersal parameters and three male reproductive success parameters were estimated from three converging MCMC sampling chains (R-hat < 1.1) (Table 4). Medians and 95% ranges of the pollen dispersal scale and shape parameters, which were correlated (Figure 2a), were estimated, and a pollen dispersal curve with confidence levels was obtained from the estimates (Figure 2b). The curve was leptokurtic and fat-tailed (*b* = 0.206) (Table 4), and the mean dispersal distance was 602 m, with a 95% range from 95 to 7,962 m. Among the three male reproductive success parameters, the size of paternal genets relative to maternal genets had a positive effect (α = 0.711), whereas both the population size of paternal genets relative to that of maternal genets (β = −0.302) and geographical separation of populations (γ = −0.575) had negative effects (Table 4). The relative genet size and separation of populations had substantial effects, since 95% ranges of these parameter estimates did not include 0, while the relative population size did not have a clear effect. Hence, the estimated parameters indicated that male reproductive success in mating between populations was lower than that within populations, and positively associated with the size of paternal genets, but negatively associated with the size of the genets’ populations.

**Table 4:** Medians and 95% confidence intervals of parameters estimated by MCMC sampling

	**Parameter**	**Median**	**(95% CI)**
*a*	scale parameter	239.709	(18.107, 1522.016)
*b*	shape parameter	0.206	(0.182, 0.257)
α	relative genet size	0.711	(0.593, 0.832)
β	relative population size	−0.302	(−0.752, 0.156)
γ	separation of population	−0.575	(−1.105, -0.021)

## Discussion

### Female reproductive success and selfing rate

Of the germinating seeds included in this study, 11.56% originated from selfing, in accordance with the known self-compatibility and mixed mating system of *M*. *stellata*[34] and congeneric species *M*. *obovata*[58,59]. Habitat fragmentation tends to decrease reproductive success less in self-compatible species than in self-incompatible species because selfing can compensate for reductions in seed production due to pollen limitation in fragmented populations [7]. However, despite the selfing potential, pollen limitations of seed production have been detected in fragmented *M*. *stellata* populations [33,60]. Hirayama et al. [33] found that manual cross-pollination results in the highest seed production rate, natural pollination the lowest, and manual self-pollination intermediate levels in fragmented populations of the species, suggesting that pollen shortage causes ovule mortality and selfing causes embryo mortality. Therefore, if outcross and/or self pollen is limited in the studied populations, pollen limitation is likely to impair their seed production and/or outcrossing opportunities.

Seed production rates increased as the population size and neighboring population size increased (Table 3). The size of focal and neighboring populations, which were indicated by the summed size of adult genets, would be positively correlated with the floral resources in the focal and surrounding populations. The rich floral resources could increase the pollinator abundance in the focal populations, leading to higher seed production rates. In the best model, the coefficient for population size was about 4.4 times higher than that of neighboring population size (Table 3), which suggests that pollinators maintained in populations are mainly dependent on floral resources in the focal populations and subsidized by those in surrounding populations. In the study site, most populations covered several hundred meters along the valleys, corresponding to the mean pollen dispersal distance (602 m). Pollination events between populations were rare (6.09%) and increases in the geographical separation of the populations significantly reduced siring success (Table 4), suggesting that most movements of pollinators occur within the populations. Thus, the populations are likely to harbor their own pollinators, partially isolating them from the other populations.

On the other hand, the local genet density had negative effects on the seed production rate, and there are two possible explanations (Table 3). First, the seed production may be reduced by the inbreeding depression due to biparental inbreeding, i.e. mating between relatives. If genetic spatial autocorrelation exists in populations, biparental inbreeding would occur, especially in the area where local genet density is high, since the pollen dispersal is limited in *M*. *stellata* as shown in this study. Under such circumstances, the opportunity to mate with relatives would increase and thus decrease the seed production rate due to inbreeding depression. However, our previous study did not detect significant spatial autocorrelation among adult genets [45], and therefore increased biparental inbreeding with increasing local genet density is not supported. Second, local competition for pollinators among neighboring plants could explain the negative effect of local density on seed production. Although it is unusual for local plant density to be negatively correlated with female reproductive success [61,62], there are a few known examples of large plant aggregations increasing competition for pollinators [15,63]. In spite of the occurrence of pollen dispersal over several hundred meters, the leptokurtic dispersal curve indicates that most pollination events occurred within about 50 m, corresponding to the scale of the local genet density (Figure 2). Thus, pollinator movements seem to be restricted to the local range, and limited availability of pollinators within the range is likely to result in the competition among the local genets.

In a previous analysis of hierarchical variation in the proportion of *M*. *stellata* seeds derived from selfing found, the selfing rate did not significantly differ among populations, but did vary among genets within populations [34]. Notwithstanding large variation in the selfing rate among genets, in the present study it was negatively correlated with local genet density. In accordance with our findings, there are tendencies for the selfing rate of various species to decrease with increases in both the local plant density and population size [2,64-66]. Van Treuren et al. [67], in particular, found that selfing in *Salvia pratensis* was promoted by low plant density, but there was no correlation between the selfing rate and population size. A low density of neighboring genets is likely to result in outcross pollen limitation and a high frequency of selfing, which can partly compensate for reductions in seed production.

The maternal genet size had negative effects on the seed production rates and positive effects on the selfing rate. This result conflicts with the expectation that the large plants have ample resources to produce large number of seeds and have large floral display to increase pollinator visitation, and thus have high seed production rate [68,69] and high outcrossing rate [70,71]. The result, however, suggests that larger genets with more flowers are frequently received geitonogamous pollination, and thus have higher selfing rate and reduces seed production [72,73]. Geitonogamous selfing is likely in *M*. *stellata* because its female-stage flowers lack rewards but morphologically mimicking male-stage flowers that produce pollinator-attracting pollen [42], and asynchronously opening within genets [74]. Self-pollination rate would increase along with the increase of maternal genet size, and some self-fertilized embryo would be aborted as Hirayama et al. [33] reported that 36 to 38% of the embryo is aborted by the self-pollination in *M*. *stellata*. The partial abortion decreases the seed production rates, and development of remaining self-fertilized embryos would increase the selfing rate.

In previous research [39], however, we found that the maternal genet size and the floral display size of neighboring genets were both positively correlated with their female reproductive success in population Y. This result is inconsistent with our present study. Thus, the effects of the maternal genet size and local genet density on female reproductive success seem to vary among populations and among years, and further research is needed to elucidate more fully the relationships among the variables and to identify more clearly the influential factors.

### Pollen flow and male reproductive success

Owing to the low value of cryptic gene flow (0.025 = 1 – 0.99992^306^), the incorrect assignment of genets within sampled candidates as pollen donors, pollen parents of nearly all examined offspring were identified amongst adult genets within the study area. The estimated shape parameter (*b* = 0.21) of the pollen dispersal kernel is smaller than previously reported values for other insect-pollinated tree species [21,32,55,56,75-78]. These comparisons suggest that the dispersal kernel of *M*. *stellata* pollen is more fat-tailed than usual, indicating extreme variation in the dispersal distance, with frequent short and rare long dispersal [56]. Among various insect visitors of *M*. *obovata* flowers, flower beetles (Scarabaeidae) carry more outcross pollen, and hence pollen with higher genetic diversity, than bumblebees and small beetles [79]. Thus, some groups of flower beetles may carry pollen over during subsequent visits among flowering genets. The main pollinators of *M*. *stellata* are also reportedly beetles, of the Staphylinidae [40], but their pollination efficiency has not been evaluated. These findings suggest that the beetle pollinators of *M*. *stellata* may occasionally disperse pollen over long distances, thus explaining the observed fat-tailed dispersal pattern.

Despite the potential for pollen dispersal over long distance, male reproductive success in mating between populations was estimated to be about half (exp (γ*u*_ij_) = *e*^-0.575^ = 0.563) that of mating within populations. This suggests that the ridges between the *M. stellata* populations are topographical barriers that impede pollen flow, possibly because different environmental conditions on the ridges hinder movements of beetle pollinators between the valleys.

The frequency of pollen-mediated gene immigration often increases as population size decreases [27], and small populations of tree species reportedly receive immigrating pollen more frequently from larger populations than vice versa [80]. These patterns seem to result from the greater abundance of pollen dispersed from large populations, regardless of the siring success of individual genets. In this study, however, the male reproductive success of individual genets tended to be higher in small populations than in large populations. Movement patterns of pollinators expected from foraging theory are probably responsible for this finding [81]. In large populations with many flowering genets, pollinators are likely to forage sequentially on abundant resources within the populations and rarely leave them. In contrast, pollinators may stay for short times within small populations and frequently leave them. These foraging patterns could enhance the probability of pollen transfer between populations from genets in small populations, in accordance with observations of increased average pollination distances in fragmented populations of animal-pollinated trees [27,82].

The estimated parameter for the effect of genet size on male reproductive success was positive, suggesting that large genets tend to have higher male reproductive success. Our previous study also indicated that larger genets sired more seedlings that had established in the study area [52]. Large genets tended to have many flowers [39], and thus abundant rewards that should attract pollinators and increase male reproductive success in spite of pollen discounting due to geitonogamous selfing.

## Conclusions

The findings of this study suggest that population structure affects mating patterns and reproductive success in *M. stellata*. Population fragmentation is likely to reduce its female and male reproductive success due to associated reductions in population sizes and increases in the geographic separation of the populations.

In spite of the potential for long-distance pollen dispersal, mating between populations was rare. Genets in larger populations tend to have higher female reproductive success, but may have lower male reproductive success. Larger genets tend to have lower seed production rates and outcrossing rates, due to geitonogamy, but higher male reproductive success. In attempts to conserve rare species, only large populations with large individuals are often selected as conservation targets. However, our study suggests that individual populations may have different, size-dependent roles. In order to conserve *M. stellata* populations, not only large populations but also small populations should be considered to maintain seed reproduction and gene flow between local populations, respectively.

## Competing interests

The authors declare that they have no competing interests.

## Authors’ contributions

SS participated in the design of the study, sample collection, genetic analyses and drafted the manuscript. TN contributed to the statistical analyses and interpretation of the results. NT collaborated in the study design and sample collection. All authors read and approved the final manuscript.

## Supplementary Material

Additional file 1 **Population size and number of offspring sired by each of the examined *Magnolia stellata *populations.** Click here for file
