# Supplementary material for: Pollen flow and effects of population structure on selfing rates and female and male reproductive success in fragmented Magnolia stellata populations
Source: BMC Ecol. 2013 Mar 22;13:10. doi: 10.1186/1472-6785-13-10 (PMC3670206; doi:10.1186/1472-6785-13-10)
Supplement: Additional file 1 — Population size and number of offspring sired by each of the examined Magnolia stellata populations. [file 1472-6785-13-10-S1.docx]

Additional file 1. Population size and number of offspring sired by each of the examined *Magnolia stellata* populations.

| Sink population of pollen | Population size | *N* | Source population of pollen (%) | | | | | | | | | | | | | | | | Total pollen immigrate (%) | |
| --- | --- | --- | --- | --- | --- | --- | --- | --- | --- | --- | --- | --- | --- | --- | --- | --- | --- | --- | --- | --- |
|  |  |  | Y | | T | | A | | B | | C | | D | | E | | F | |  |  |
| Y | 85 | 167 | 155 | - | 9 | (5.39) | 1 | (0.60) | 2 | (1.20) | 0 | (0.00) | 0 | (0.00) | 0 | (0.00) | 0 | (0.00) | 12 | (7.19) |
| T | 97 | 137 | 2 | (1.46) | 129 | - | 4 | (2.92) | 1 | (0.73) | 0 | (0.00) | 0 | (0.00) | 1 | (0.73) | 0 | (0.00) | 8 | (5.84) |
| A | 46 | 67 | 4 | (5.97) | 0 | (0.00) | 61 | - | 1 | (1.49) | 0 | (0.00) | 0 | (0.00) | 1 | (1.49) | 0 | (0.00) | 6 | (8.96) |
| B | 16 | 30 | 0 | (0.00) | 0 | (0.00) | 0 | (0.00) | 30 | - | 0 | (0.00) | 0 | (0.00) | 0 | (0.00) | 0 | (0.00) | 0 | (0.00) |
| C | 23 | 83 | 0 | (0.00) | 2 | (2.41) | 0 | (0.00) | 0 | (0.00) | 81 | - | 0 | (0.00) | 0 | (0.00) | 0 | (0.00) | 2 | (2.41) |
| F | 4 | 9 | 0 | (0.00) | 2 | (22.22) | 0 | (0.00) | 0 | (0.00) | 0 | (0.00) | 0 | (0.00) | 0 | (0.00) | 7 | - | 2 | (22.22) |

**Abbreviations**: *N*, the number of offspring analyzed in each population. Meshed cell indicate the within population pollen flow.
